# Preparedness for self-isolation or quarantine and lockdown in South Africa: results from a rapid online survey

**DOI:** 10.1186/s12889-021-10628-9

**Published:** 2021-03-23

**Authors:** Sibusiso Sifunda, Tholang Mokhele, Thabang Manyaapelo, Natisha Dukhi, Ronel Sewpaul, Whadi-Ah Parker, Saahier Parker, Inbarani Naidoo, Sean Jooste, Shandir Ramlagan, Razia Gaida, Musawenkosi Mabaso, Khangelani Zuma, Priscilla Reddy

**Affiliations:** 1 Human Sciences Research Council, Human and Social Capabilities Research Division, Private Bag X41, Pretoria, 0001 South Africa; 2 School of Public Health, University of the Witwatersrand, Johannesburg, South Africa

**Keywords:** COVID-19, Pandemic, Preparedness, Self-isolation, Quarantine, South Africa

## Abstract

**Background:**

The World Health Organization (WHO) declared the COVID-19 pandemic a public health emergency of international concern. South Africa, like many other countries, initiated a multifaceted national response to the pandemic. Self-isolation and quarantine are essential components of the public health response in the country. This paper examined perceptions and preparedness for self-isolation or quarantine during the initial phase of the pandemic in South Africa.

**Methods:**

The analysis used data obtained from an online quantitative survey conducted in all nine provinces using a data-free platform. Descriptive statistics and multivariable logistic regression models were used to analyse the data.

**Results:**

Of 55,823 respondents, 40.1% reported that they may end up in self-isolation or quarantine, 32.6% did not think that they would and 27.4% were unsure. Preparedness for self-isolation or quarantine was 59.0% for self, 53.8% for child and 59.9% for elderly. The odds of perceived possibility for self-isolation or quarantine were significantly higher among Coloureds, Whites, and Indians/Asians than Black Africans, and among those with moderate or high self-perceived risk of contracting COVID-19 than those with low risk perception. The odds were significantly lower among older age groups than those aged 18–29 years, and those unemployed than fully employed. The odds of preparedness for self-isolation or quarantine were significantly less likely among females than males. Preparedness for self, child and elderly isolation or quarantine was significantly more likely among other population groups than Black Africans and among older age groups than those aged 18–29 years. Preparedness for self, child and elderly isolation or quarantine was significantly less likely among those self-employed than fully employed and those residing in informal dwellings than formal dwellings. In addition, preparedness for self-isolation or quarantine was significantly less likely among those with moderate and high self-perceived risk of contracting COVID-19 than low risk perception.

**Conclusion:**

The findings highlight the challenge of implementing self-isolation or quarantine in a country with different and unique social contexts. There is a need for public awareness regarding the importance of self-isolation or quarantine as well as counter measures against contextual factors inhibiting this intervention, especially in impoverished communities.

**Supplementary Information:**

The online version contains supplementary material available at 10.1186/s12889-021-10628-9.

## Background

In December 2019, an outbreak of a pneumonia-like illness caused by a novel coronavirus first emerged in Wuhan, Hubei Province, China and subsequently spread rapidly across the rest of the world [1]. This virus is now formally referred to as severe acute respiratory syndrome coronavirus 2 (SARS-CoV-2) named ‘coronavirus disease 2019’ (COVID-19). On 30th January 2020, the World Health Organization (WHO) declared Covid-19 a public health emergency of international concern and about a month later, on March 2020, WHO reclassified COVID-19 as a global pandemic [2]. By July 2020, all continents reported confirmed COVID-19 cases and this novel coronavirus had spread to more than 215 countries including Africa [3].

Currently, immuno-compromised individuals, the elderly and those with pre-existing health conditions such as diabetes, hypertension and obesity appear to be most affected by COVID-19 [4–7]. Since the emergence of COVID-19, many countries in Africa including South Africa are facing a catastrophe due to increasing numbers of cases and fragile health systems [8]. South Africa currently has the highest number of confirmed COVID-19 cases on the African continent [9].

In the absence of specific therapeutics, and during vaccine development and initial roll-out, the world has relied on public health and socio-behavioural measures to reduce the spread of COVID-19 [10]. These interventions include social approaches such as quarantine, isolation and lockdown. Quarantine refers to the restriction of movement of people who may have been exposed to a contagious disease or were in contact with an infected person, and is the oldest containment strategy [10]. Isolation refers to the separation of people who are known to be infected with a contagious disease from those who are uninfected in order to limit viral shedding and thereby control the spread of the disease [11]. Lockdown or community containment is a full mandatory quarantine or non-mandatory “stay at home order” whereby events and businesses are halted, and the public are requested to remain at home in an effort to restrict mass gatherings and thereby restrict viral transmission [12].

According to the WHO pandemic guidelines, individuals play an important role in the pandemic preparedness and they need to be aware of the importance of their role [13]. The behaviours of individuals, which are associated with their risk perceptions, have a direct impact on the trajectory of an infectious disease outbreak [14]. For example, during the SARS, Middle East respiratory syndrome and Ebola outbreaks, public health measures, such as quarantine and isolation, were also rigorously implemented to disrupt human-to-human transmission and to strengthen control measures [15, 16]. In most instances the intensive quarantine and isolation measures curbed further transmission [16, 17]. While quarantine and isolation orders during the Ebola outbreak led to challenges in access to food, water and sanitation, they proved successful in reducing disease transmission [18].

In response to the COVID-19 pandemic in South Africa, the government declared a state of national disaster on 15th March 2020 [19]. A nationwide lockdown was declared 2 weeks later on the 27th of March 2020, which was effective for 21 days, and then later extended to the end of April 2020 [20]. Only essential services including food production, distribution and sales; and pharmaceutical and medical services were permitted to operate [21]. During this period the government raised awareness about the importance of quarantine and isolation measures for reducing the transmission of the virus. As the threat of COVID-19 continues to grow, understanding the population’s preparedness for self-isolation or quarantine is vital for informing policy and future interventions in the fight against this pandemic. This paper therefore examined perceptions and preparedness for self-isolation or quarantine and preparations for lockdown during the initial phase of the pandemic in South Africa.

## Methods

### Data source

The data used in the analysis is based on the Human Sciences Research Council’s (HSRC) COVID-19 rapid online survey. The study was conducted using the data free Moya Messaging platform (Moya Messaging App, South Africa) [22], which allowed anyone with a mobile phone to receive and respond to the survey, without incurring data costs. In addition, information about the survey was distributed on various social media platforms and on the HSRC website. The communication alerts included an invitation with a link, which when clicked on, directed potential respondents to the survey. The online survey was conducted from 27 March 2020 to 2 April 2020, which coincided with the first 7 days of the lockdown period for COVID-19. All adults (18 years and older) residing in South Africa, regardless of race, sex or nationality were eligible to participate. Location in South Africa was restricted by internet protocol address to ensure that only residents in South Africa could complete the survey.

A structured questionnaire was developed for this study, which was based broadly on COVID-19 surveys in other countries as well as a review of the literature [23, 24]. The online questionnaire was only administered to consenting participants. The self-administered questionnaire (attached as an additional file) consisted of items on socio-demographic information, knowledge and infection control measures regarding COVID-19, hygiene practices, preparedness for self-isolation/quarantine, preparedness for lockdown and the use of social media in accessing information on COVID-19. The questionnaire had 48 items, and this paper focused on questions relating to perceptions and preparedness for self-isolation or quarantine and lockdown.

### Measures

#### Primary outcome variables

Perceptions for self-isolation or quarantine possibility were assessed using the question “Do you think you may end up in a situation of self-isolation or quarantine?” with response options 1 = yes, 2 = no and 3 = don’t know dichotomized into a binary outcome (yes = 1 and no/don’t know = 0). Preparedness for self-isolation, child-isolation and elderly-isolation was assessed using three questions. The first question was “If quarantine / self-isolation should become necessary, does your home have a separate space for you to do so?” with response options 1 = yes, 2 = no, 3 = don’t know and 4 = n/a – I live alone dichotomized into a binary outcome (yes = 1 and no/don’t know/I live alone = 0). The second question was “If there are children in the home needing self-quarantine, would you be able to separate them from the rest of the family? (aged 0-14)” with response options being 1 = yes, 2 = no and 3 = don’t know dichotomized into a binary outcome (yes = 1 and no/don’t know = 0). The third question was “If there are elderly members in the home needing self-quarantine, would you be able to separate them from the rest of the family?” with response options being 1 = yes, 2 = no and 3 = don’t know dichotomized into a binary outcome (yes = 1 and no/don’t know = 0).

#### Explanatory variables

This included socio-demographic variables such as age group in years (18–29, 30–39, 40–49, 50–59, 60 and older), sex (male, female), race group (Black African, Coloured, White, Indian/Asian), employment status (employed full time, employed informally/part time, student, unemployed, self-employed), and dwelling type (formal dwelling or informal dwelling). The racial classification used was based on Statistics South Africa’s guidelines for data collection in all national surveys including the census. Reporting by racial classification in South Africa, with a long history of colonialism and racial segregation, provides data to inform government policies that include addressing historical inequalities and racial disparities. These racial classifications are only used as risk markers for the systemic and structural disadvantages and not as health risk factors.

Self-perceived risk of contracting coronavirus was assessed using the question “How do you rate your personal risk of contracting COVID-19?” with response options being 1 = very high risk, 2 = high risk, 3 = moderate risk, 4 = low risk and 5 = very low risk. These responses were recoded into 1 = low risk (very low risk, and low risk), 2 = moderate risk and 3 = high risk (very high risk and high risk).

### Data analysis

Data were analysed using Stata version 15.0 [25]. The data were benchmarked using the South Africa’s 2019 mid-year adult population (≥ 18 years) estimates by age, race, sex and province to allow for the generalizability of findings to the rest of the country. Benchmarking, also known as post-stratification, is the process of adjusting the weights so that they sum to the known population totals. The “svy” command was used to incorporate benchmarking weights into the analysis [26]. Descriptive statistics (frequencies and percentages) were used to summarize the primary outcome measures by socio-demographic characteristics. Pearson’s chi-square test was used to compare differences between categorical variables. Multivariable logistic regression models were fitted to determine the factors associated with 1) perceptions for self-isolation or quarantine possibility and 2) preparedness for each of self-isolation or quarantine, child-isolation or quarantine and elderly-isolation or quarantine. Odds Ratios (OR) and 95% Confidence Intervals (CIs) with a *p* value less than 0.05 were used to ascertain the level of statistical significance. The map was created using ArcGIS 10.7.1 [27].

## Results

### Demographic characteristics of respondents

A total of 55,823 individuals responded to the online survey. Table 1 shows socio-demographic characteristics of the study sample. Just above a half of the respondents were females, the majority were Black African, over a third were aged 18–29 years, and almost half were unemployed. The majority resided in informal dwellings and the Gauteng, KwaZulu-Natal and Western Cape provinces constituted the highest proportion of participants.

**Table 1:** Socio-demographic characteristics of the study sample

Variables	n	%	95% CI
**Sex**			
Male	16,365	47.9	46.7–49.1
Female	34,927	52.1	50.9–53.3
**Age group (years)**			
18–29	9519	31.5	30.4–32.6
30–39	13,665	25.9	24.9–26.8
40–49	13,496	17.0	16.2–17.8
50–59	10,956	12.1	11.4–12.8
60–69	6054	8.1	7.4–8.8
70+	1927	5.5	4.5–6.7
**Race group**			
Black African	9083	78.4	77.8–78.9
Coloured	4688	9.0	8.7–9.4
White	36,878	9.6	9.4–9.9
Indian/Asian	4016	3.0	2.8–3.1
**Employment status**			
Employed full time	29,003	49.3	48.1–50.5
Employed informal/part time	3984	7.8	7.2–8.4
Student	3116	11.6	10.9–12.4
Unemployed	8861	20.3	19.2–21.4
Self employed	10,625	10.9	10.1–11.8
**Dwelling type**			
Formal dwelling	52,971	95.6	95.0–96.1
Informal dwelling	606	4.4	3.9–5.0
**Province**			
Eastern Cape	2617	10.5	9.8–11.2
Free State	1339	4.9	4.4–5.3
Gauteng	25,553	28.0	27.3–28.8
KwaZulu-Natal	6734	18.3	17.5–19.1
Limpopo	1116	9.4	8.5–10.3
Mpumalanga	1031	7.6	6.9–8.4
North West	1045	6.7	6.0–7.5
Northern Cape	519	2.1	1.9–2.4
Western Cape	15,869	12.4	12.0–12.9

Sub-totals are not always equal to the overall total due to non-response or missing data

*CI* Confidence Interval

### Percieved possibility of self-isolation or quarantine

**Fig. 1:**
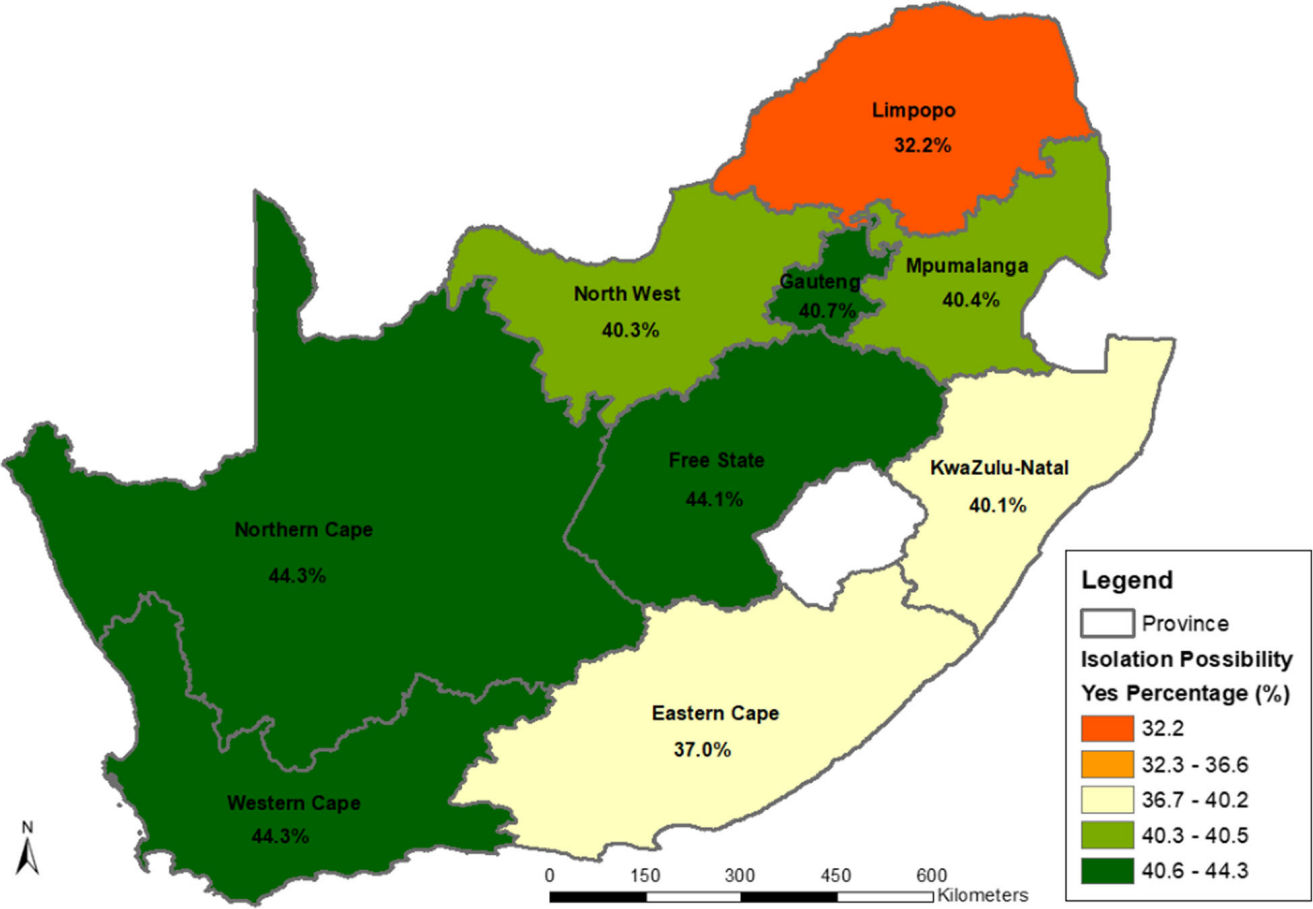
Residents who thought they might end up in a situation of self-isolation or quarantine (Own generated map, developed using ArcGIS 10.7.1, Shapefile Data Source – Municipal Demarcation Board http://www.demarcation.org.za/)

Overall, about two-fifths (40.1, 95% CI: 38.9–41.3) reported that they may end up in self-isolation or quarantine, 32.6%, (95% CI: 31.3–33.7) did not think they would end up in isolation or quarantine, while 27.4%, (95% CI: 26.2–28.6) were unsure if they would end up in this situation. There was significant variation (*p* < 0.001) in the perceived possibility for self-isolation or quarantine by province. A higher proportion of those residing in Western Cape (44.3%), Northern Cape (44.3%), Free State (44.1%) and Gauteng (40.7%) reported that they might end up in self-isolation or quarantine, and lower proportions were in reported in Limpopo (32.2%) and Eastern Cape 37.0 (Fig. 1).

Table 2 shows the perceived possibility of self-isolation or quarantine by socio-demographic characteristics. More males (40.4, 95% CI: 38.4–42.5) reported that they might end up in self-isolation or quarantine than females (39.6, 95% CI: 38.1–41.1). There was no significant difference in perceived possibility of self-isolation or quarantine between the age groups. However, perceived possibility for self-isolation or quarantine was lowest among those aged 50–59 years (36.6, 95% CI: 33.6–39.7) and 60–69 years (36.6, 95% CI: 32.6–41.4). There was a significant difference in perceived possibility for self-isolation or quarantine between the population groups (*p* < 0.001). Black Africans (38.8, 95% CI: 37.2–40.4) reported lower proportions compared to the other population groups.

**Table 2:** Residents’ responses on whether they thought they might end up in a situation of self-isolation or quarantine

Demographics	Total	Yes		No		I don’t know		***P*** value
	***n***	%	95% CI	%	95% CI	%	95% CI	
**Total**	**53,503**	**40.1**	**[38.9–41.3]**	**32.5**	**[31.3–33.7]**	**27.4**	**[26.2–28.6]**	
**Sex**								0.032
Male	15,475	40.4	[38.4–42.5]	28.7	[26.7–30.7]	30.9	[29.0–32.9]	
Female	33,729	39.6	[38.1–41.1]	26.3	[24.9–27.8]	34.1	[32.6–35.7]	
**Age group (years)**								0.157
18–29	8813	42.6	[40.4–44.8]	27.1	[25.2–29.1]	30.3	[28.3–32.4]	
30–39	13,075	40.4	[38.4–42.4]	26.8	[24.9–28.7]	32.8	[30.9–34.8]	
40–49	13,093	39.1	[36.7–41.5]	25.9	[23.9–28.2]	34.9	[32.7–37.3]	
50–59	10,678	36.6	[33.6–39.7]	28.7	[25.7–31.8]	34.7	[31.8–37.8]	
60–69	5882	36.9	[32.6–41.4]	33.4	[28.7–38.5]	29.7	[25.6–34.1]	
70+	1862	40.8	[31.1–51.2]	24.1	[15.7–35.1]	35.1	[25.4–46.2]	
**Race group**								< 0.001
Black African	7907	38.8	[37.2–40.4]	29.1	[27.5–30.6]	32.2	[30.6–33.8]	
Coloured	4380	40.1	[38.2–42.1]	22.8	[21.1–24.6]	37.1	[35.2–39.0]	
White	36,310	47.7	[46.7–48.6]	20.7	[20.0–21.5]	31.6	[30.8–32.5]	
Indian/Asian	3873	41	[38.6–43.4]	24.9	[22.6–27.3]	34.1	[31.9–36.5]	
**Employment**								0.425
Employed full time	28,198	40.6	[39.0–42.1]	26.9	[25.4–28.3]	32.6	[31.1–34.1]	
Employed informal/part time	3779	40.6	[36.4–44.9]	27.2	[22.9–31.9]	32.2	[28.4–36.4]	
Student	2937	41.5	[37.9–45.2]	26.8	[23.6–30.3]	31.7	[28.3–35.3]	
Unemployed	8138	36.2	[32.8–39.6]	29.2	[25.6–33.0]	34.6	[31.1–38.4]	
Self employed	10,341	41.2	[37.0–45.6]	27.7	[24.4–31.2]	31.1	[26.9–35.6]	
**Dwelling type**								0.110
Formal dwelling	52,890	40.2	[39.0–41.5]	32.2	[31.0–33.5]	27.5	[26.3–28.8]	
Informal dwelling	604	37.4	[31.8–43.3]	38.4	[32.7–44.5]	24.2	[19.4–29.8]	
**Province**								< 0.001
Eastern Cape	2468	37	[33.0–41.3]	32.8	[28.8–37.0]	30.2	[26.3–34.4]	
Free State	1258	44.1	[38.4–50.0]	24.0	[19.5–29.2]	31.9	[26.5–37.7]	
Gauteng	24,549	40.7	[39.3–42.1]	32.4	[31.0–33.8]	26.9	[25.6–28.3]	
KwaZulu-Natal	6403	40.1	[37.2–43.0]	34.9	[32.3–37.6]	25.0	[22.7–27.5]	
Limpopo	1020	32.2	[26.3–38.8]	30.2	[24.6–36.4]	37.6	[31.0–44.6]	
Mpumalanga	960	40.4	[34.4–46.7]	33.8	[28.1–40.1]	25.8	[20.7–31.6]	
North West	973	40.3	[33.6–47.4]	29.5	[22.0–38.4]	30.2	[24.2–36.9]	
Northern Cape	478	44.3	[36.2–52.7]	34.8	[27.4–42.9]	20.9	[14.8–28.7]	
Western Cape	15,394	44.3	[42.6–46.1]	34.4	[32.7–36.0]	21.3	[19.8–22.9]	

Sub-totals are not always equal to the overall total due to non-response or missing data

*CI* Confidence Interval

There was no significant variation in perceived possibility for self-isolation or quarantine by employment status. However, perceived possibility for self-isolation or quarantine was lowest among the unemployed (36.2, 95% CI: 32.8–39.6). There was also no significant variation by dwelling type. However, perceived possibility for self-isolation or quarantine was lower among those residing in informal dwellings (37.4, 95% CI: 31.8–43.3).

**Fig. 2:**
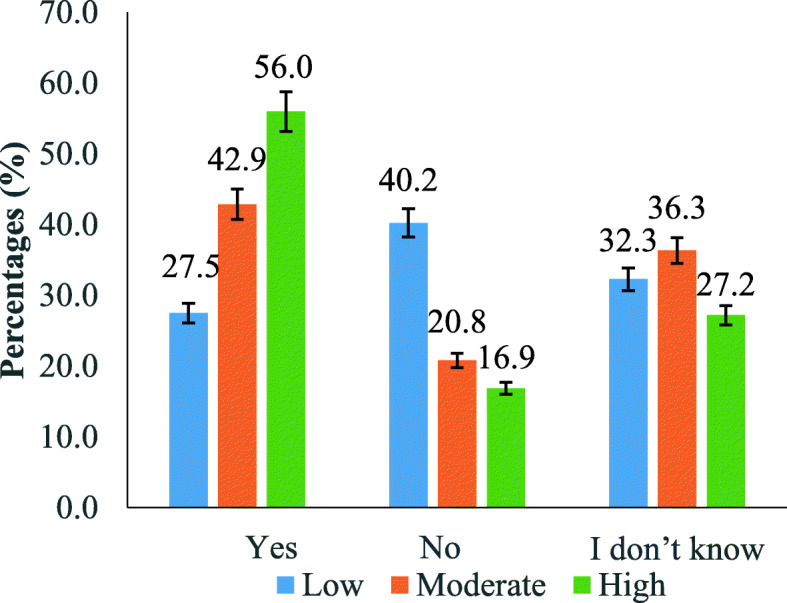
Proportion of residents who thought they might end up in a situation of self-isolation or quarantine by risk perception

Figure 2 shows that perceived possibility of self-isolation or quarantine was higher among those with moderate- and high-risk perception of contracting COVID-19. The highest proportions were reported among those with high risk perception at 56.0% and the lowest proportion among those with low risk perception at 27.5%.

Table 3 shows a multivariable logistic model of factors associated with perceived possibility for self-isolation or quarantine. The odds of perceived possibility for self-isolation or quarantine were significantly higher among individuals who identified as Coloured (OR = 1.17, 95% CI: 1.04–1.31, *p* = 0.007), White (OR = 1.92, 95% CI: 1.66–2.22, *p* < 0.001) and Indian/Asian (OR = 1.23, 95% CI: 1.07–1.41, *p* = 0.003) than among those who identified as Black African, and among those who had moderate (OR = 2.01, 95% CI: 1.80–2.26, *p* < 0.001) and high (OR = 3.60, 95% CI: 3.14–4.11, *p* < 0.001) self-perceived risk of contracting COVID-19 than low risk. The odds of perceived possibility for self-isolation or quarantine were significantly lower among those aged 30–39 years (OR = 0.85, 95% CI: 0.74–0.97, *p* = 0.021), 40–49 years (OR = 0.78, 95% CI: 0.66–0.91, *p* = 0.002), 50–59 years (OR = 0.65, 95% CI: 0.54–0.78, *p* < 0.001), and 60 years and older (OR = 0.61, 95% CI: 0.46–0.79, *p* < 0.001) than those aged 18–29 years, and among the unemployed (OR = 0.84, 95% CI: 0.71–0.99, *p* = 0.040) than those employed full time.

**Table 3:** Factors associated with perception on self-isolation or quarantine possibility

	OR	95% CI	***p*** value
**Characteristics**			
**Sex**			
Male (Ref)			
Female	0.93	[0.83–1.03]	0.157
**Race group**			
Black (Ref)			
Coloured	1.17	[1.04–1.31]	0.007
White	1.92	[1.66–2.22]	< 0.001
Indian/Asian	1.23	[1.07–1.41]	0.003
**Age group**			
18–29 (Ref)			
30–39	0.85	[0.74–0.97]	0.021
40–49	0.78	[0.66–0.91]	0.002
50–59	0.65	[0.54–0.78]	< 0.001
60+	0.61	[0.46–0.79]	< 0.001
**Employment**			
Employed full time (Ref)			
Employed informal/part time	0.99	[0.81–1.20]	0.900
Student	1.04	[0.85–1.26]	0.718
Unemployed	0.84	[0.71–0.99]	0.040
Self employed	1.05	[0.88–1.26]	0.572
**Knowledge score**			
Low (Ref)			
Moderate	1.00	[0.78–1.28]	0.982
High	0.98	[0.77–1.24]	0.838
**Risk perception**			
Low (Ref)			
Moderate	2.01	[1.80–2.26]	< 0.001
High	3.60	[3.14–4.11]	< 0.001
**Dwelling type**			
Formal dwelling (Ref)			
Informal dwelling	0.77	[0.58–1.01]	0.061
Constant	0.46	[0.35–0.60]	< 0.001

*CI* Confidence Interval, *OR* Odds Ratio

### Preparedness for self-isolation or quarantine

Of 52,493 respondents, 59.0% (95% CI: 57.8–60.3) reported that they were prepared for self-isolation or quarantine, 53.8% (95% CI: 52.5–55.0) for child-isolation or quarantine and 59.9% (95% CI: 58.7–61.1]) for elderly-isolation or quarantine. Table 4 shows preparedness for self-isolation, child-isolation and elderly-isolation by socio-demographic characteristics. Preparedness for self-isolation or quarantine and child-isolation or quarantine was significantly higher among males than females, with *p* = 0.001 and *p* = 0.050 respectively. Preparedness for self, child and elderly isolation or quarantine increased significantly with the age of the respondent. Furthermore, preparedness for self, child, and elderly isolation or quarantine were significantly higher among the other race groups than among Black Africans. Preparedness for self, child, and elderly isolation or quarantine were highest among the self-employed and lowest among students. In addition, preparedness for self, child, and elderly isolation or quarantine was significantly higher among those residing in formal dwellings than informal dwellings. There were significant variations in preparedness for self, child, and elderly isolation or quarantine by province.

**Table 4:** Preparedness for self-isolation, child-isolation and elderly-isolation

Demographics	Total	Self-isolation			Child-isolation			Elderly-isolation		
	***n***	%	95% CI	***p*** value	%	95% CI	***p*** value	%	95% CI	***p*** value
**Total**	**52,493**	**59.0**	**[57.8–60.3]**		**53.8**	**[52.5–55.0]**		**59.9**	**[58.7–61.1]**	
**Sex**				0.001			0.050			0.818
Male	15,233	61.3	[59.3–63.3]		55.2	[53.1–57.2]		60.1	[58.0–62.1]	
Female	33,020	57.0	[55.5–58.6]		52.5	[51.0–54.1]		59.8	[58.3–61.2]	
**Age group (years)**				< 0.001			< 0.001			< 0.001
18–29	8728	47.2	[45.0–49.4]		42.9	[40.7–45.1]		51.5	[49.2–53.7]	
30–39	12,947	50.8	[48.7–52.9]		47.4	[45.3–49.5]		52.7	[50.6–54.8]	
40–49	12,962	63.7	[61.3–66.1]		61.2	[58.8–63.6]		62.5	[60.1–64.9]	
50–59	10,374	75.6	[72.7–78.3]		68.8	[65.8–71.6]		71.2	[68.2–74.0]	
60–69	5637	79.0	[75.1–82.5]		68.1	[63.6–72.3]		75.0	[70.6–79.0]	
70+	1749	76.8	[65.8–85.0]		64.6	[53.6–74.2]		82.3	[72.1–89.3]	
**Race group**				< 0.001			< 0.001			< 0.001
Black African	7858	55.0	[53.4–56.7]		51.6	[50.0–53.2]		57.0	[55.4–58.6]	
Coloured	4343	64.1	[62.2–65.9]		57.5	[55.5–59.4]		63.8	[61.9–65.6]	
White	35,473	77.8	[77.1–78.5]		63	[62.1–63.9]		74.2	[73.5–75.0]	
Indian/Asian	3840	75.3	[73.4–77.1]		64	[61.7–66.2]		71.7	[69.6–73.7]	
**Employment**				< 0.001			< 0.001			< 0.001
Employed full time	27,783	59.0	[57.4–60.5]		54.8	[53.2–56.4]		59.0	[57.4–60.5]	
Employed informal/part time	3721	50.7	[46.3–55.1]		45.1	[40.7–49.6]		51.6	[47.2–56.0]	
Student	2899	49.0	[45.3–52.8]		42.4	[38.7–46.2]		50.9	[47.1–54.6]	
Unemployed	7890	60.7	[57.2–64.1]		55.0	[51.6–58.4]		65.3	[62.1–68.4]	
Self employed	10,095	72.5	[68.0–76.6]		65.2	[60.7–69.3]		71.0	[66.5–75.1]	
**Dwelling type**				< 0.001			< 0.001			< 0.001
Formal dwelling	51,887	60.6	[59.3–61.8]		54.9	[53.6–56.2]		61.1	[59.9–62.4]	
Informal dwelling	599	25.8	[20.8–31.5]		30.6	[25.3–36.5]		34.3	[28.9–40.2]	
**Province**				< 0.001			0.001			0.002
Eastern Cape	2429	59.9	[55.5–64.0]		54.5	[50.2–58.8]		60.4	[56.2–64.4]	
Free State	1246	53.4	[47.5–59.2]		51.9	[46.0–57.8]		54.7	[48.8–60.5]	
Gauteng	24,132	63.0	[61.6–64.4]		55.1	[53.6–56.6]		61.0	[59.6–62.5]	
KwaZulu-Natal	6297	54.6	[51.7–57.4]		47.4	[44.5–50.3]		57.6	[54.8–60.4]	
Limpopo	1011	64.0	[57.6–69.8]		63.9	[58.2–69.3]		69.5	[63.9–74.6]	
Mpumalanga	953	56.9	[50.6–63.0]		58.3	[52.0–64.3]		55.8	[49.5–62.0]	
North West	962	54.1	[46.5–61.5]		52.9	[45.3–60.5]		59.1	[51.2–66.5]	
Northern Cape	477	52.6	[44.2–60.8]		48.8	[40.6–57.1]		58.6	[50.2–66.6]	
Western Cape	14,986	59.1	[57.3–60.8]		51.7	[49.9–53.5]		58.6	[56.8–60.4]	

Sub-totals are not always equal to the overall total due to non-response or missing data

*CI* Confidence Interval

Table 5 shows three multivariable logistic regression models of factors associated with preparedness for self, child, and elderly isolation or quarantine. Preparedness for self-isolation or quarantine was significantly less likely among females (OR = 0.86, 95% CI: [0.77–0.97], *p* = 0.010) than males. Preparedness for child-isolation or quarantine was significantly less likely among those employed informally/part time than full time (OR = 0.77, 95% CI: 0.64–0.94, *p* = 0.008) and preparedness for elderly-isolation or quarantine was significantly less likely among those employed informally/part time than full time (OR = 0.81, 95% CI: 0.67–0.98, *p* = 0.034). Preparedness for self, child and elderly isolation or quarantine were significantly less likely among those residing in informal dwellings than formal dwellings with OR = 0.33, 95% CI: 0.24–0.45, *p* < 0.001; OR = 0.48, 95% CI: 0.36–0.63, *p* < 0.001 and OR = 0.44, 95% CI: 0.34–0.57 respectively. In addition, those who perceived the risk of contracting COVID-19 as moderate (OR = 0.83, 95% CI: 0.72–0.94, *p* = 0.004) and high (OR = 0.59 95% CI: 0.51–0.68, *p* < 0.001) were significantly less likely to be prepared for self-isolation or quarantine than those with low risk perception. Similar trends were observed with regards to preparedness for child and elderly isolation or quarantine.

**Table 5:** Factors associated with preparedness for self-isolation, child-isolation and elderly-isolation

	Self-isolation			Child-isolation			Elderly-isolation		
	OR	95% CI	***p*** value	OR	95% CI	***p*** value	OR	95% CI	***p*** value
**Characteristics**									
**Sex**									
Male (Ref)									
Female	0.86	[0.77–0.97]	0.010	0.95	[0.85–1.05]	0.319	1.04	[0.93–1.15]	0.505
**Race group**									
Black (Ref)									
Coloured	1.18	[1.05–1.33]	0.005	1.04	[0.93–1.16]	0.538	1.10	[0.99–1.23]	0.081
White	1.6	[1.36–1.88]	< 0.001	1.00	[0.86–1.16]	0.981	1.26	[1.09–1.46]	0.002
Indian/Asian	1.84	[1.61–2.10]	< 0.001	1.27	[1.11–1.45]	0.001	1.46	[1.28–1.67]	< 0.001
**Age group**									
18–29 (Ref)									
30–39	1.1	[0.96–1.27]	0.168	1.12	[0.98–1.29]	0.104	1.00	[0.87–1.14]	0.953
40–49	1.74	[1.49–2.03]	< 0.001	1.87	[1.61–2.18]	< 0.001	1.41	[1.21–1.64]	< 0.001
50–59	2.89	[2.38–3.51]	< 0.001	2.54	[2.12–3.05]	< 0.001	2.01	[1.67–2.43]	< 0.001
60+	3.3	[2.36–4.60]	< 0.001	2.5	[1.88–3.32]	< 0.001	2.7	[1.98–3.68]	< 0.001
**Employment**									
Employed full time (Ref)									
Employed informal/part time	0.83	[0.68–1.00]	0.055	0.77	[0.64–0.94]	0.008	0.81	[0.67–0.98]	0.034
Student	0.99	[0.82–1.20]	0.945	0.86	[0.71–1.04]	0.122	0.91	[0.76–1.10]	0.345
Unemployed	0.92	[0.77–1.10]	0.37	0.97	[0.82–1.14]	0.673	1.15	[0.97–1.35]	0.104
Self employed	1.41	[1.10–1.80]	0.006	1.29	[1.05–1.59]	0.017	1.4	[1.11–1.77]	0.005
**Knowledge score**									
Low (Ref)									
Moderate	0.98	[0.77–1.25[	0.875	0.99	[0.78–1.26]	0.948	1.19	[0.93–1.52]	0.161
High	0.99	[0.79–1.25]	0.965	0.99	[0.79–1.25]	0.937	1.14	[0.90–1.44]	0.286
**Risk perception**									
Low (Ref)									
Moderate	0.83	[0.72–0.94]	0.004	0.69	[0.61–0.78]	< 0.001	0.81	[0.71–0.91]	0.001
High	0.59	[0.51–0.68]	< 0.001	0.61	[0.54–0.70]	< 0.001	0.62	[0.54–0.71]	< 0.001
**Might end up in self-isolation or quarantine**									
No (Ref)									
Yes	1.24	[1.12–1.38]	< 0.001	0.99	[0.89–1.10]	0.891	1.11	[1.00–1.24]	0.042
**Dwelling type**									
Formal dwelling (Ref)									
Informal dwelling	0.33	[0.24–0.45]	< 0.001	0.48	[0.36–0.63]	< 0.001	0.44	[0.34–0.57]	< 0.001
Constant	1.15	[0.88–1.49]	0.315	1.12	[0.86–1.47]	0.394	1.1	[0.84–1.45]	0.474

*CI* Confidence Interval, *OR* Odds Ratio

Preparedness for self-isolation or quarantine was significantly more likely among individuals who identified themselves as Coloured (OR = 1.18 95% CI: 1.05–1.33, *p* = 0.005), White (OR = 1.60, 95% CI: 1.36–1.88, *p* < 0.001) and Indian or Asian (OR = 1.84 95% CI: 1.61–2.10, *p* < 0.001) than those who identified as Black African. Preparedness for child isolation or quarantine was significantly more likely among Indian/Asian (OR = 1.27, 95% CI: 1.11–1.45, *p* = 0.001) than Black African participants, and preparedness for elderly isolation or quarantine was significantly more likely among White (OR = 1.26, 95% CI: 1.09–1.46, *p* = 0.002) and Indian/Asian (OR = 1.46 95% CI: 1.28–1.67, *p* < 0.001) than Black African participants. Those aged 40–49 years (OR = 1.74, 95% CI: 1.49–2.03, *p* < 0.001), 50–59 years (OR = 2.89, 95% CI: 2.38–3.51, *p* < 0.001), and 60 years and older (OR = 3.30, 95% CI: 2.36–4.60, *p* < 0.001) were significantly more likely to be prepared to self-isolate or quarantine than those aged 18–29 years. Similar trends were observed for preparedness for child and elderly isolation or quarantine. Preparedness for self, child and elderly isolation or quarantine were significantly more likely among the self-employed than those employed fulltime (OR = 1.41, 95% CI: 1.10–1.80, *p* = 0.006; OR = 1.29, 95% CI: 1.05–1.59, *p* = 0.017 and OR = 1.40, 95% CI: 1.11–1.77, *p* = 0.005 respectively). Preparedness for self, child and elderly isolation or quarantine were significantly more likely among those residing in informal dwellings than formal dwellings with OR = 0.33 95% CI: 0.24–0.45, *p* < 0.001; OR = 0.48 95% CI: 0.36–0.63, *p* < 0.001 and OR = 0.44 95% CI: 0.34–0.57, *p* < 0.001 respectively.

### Preparation for the hard “Stay at home” lockdown

**Fig. 3:**
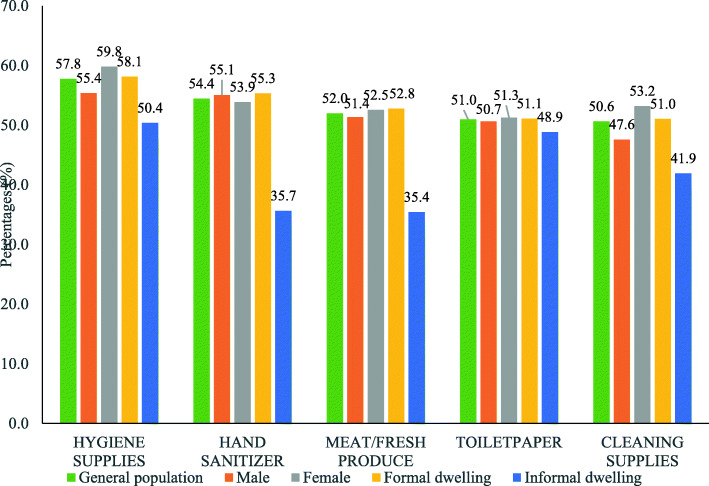
Responses on what the residents bought MORE in preparation for the lock-down period by sex and dwelling type

Figure 3 shows that the five most commonly purchased products in preparation for lockdown were hygiene supplies/products (57.8%), hand sanitizer (54.4%), meat/fresh produce (52.0%), toilet paper (51.0%) and cleaning supplies (50.6%) (Fig. 3). Approximately 29% purchased luxury items/snacks, while 24.6% indicated that they did not purchase any products in preparation for the lockdown. Both males and females followed similar trends with females reporting higher prevalence of purchasing hygiene supplies/products (57.8%) and cleaning supplies (53.2%). Males reported a higher prevalence of purchasing hand sanitizers (55.1%). Residents living in informal dwellings reported different purchasing patterns from the general population, in that 50.4% purchased hygiene supplies/products, 48.9% toilet paper, 41.9% cleaning supplies, 35.7% hand sanitizer and 35.4% purchased meat/fresh produce.

**Fig. 4:**
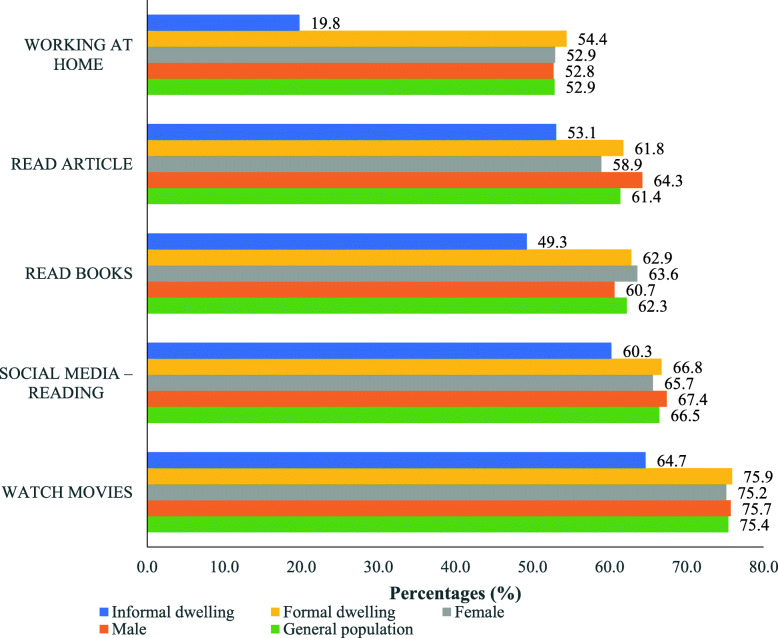
Responses on how the residents would occupy their time during 21 days of lockdown

When asked how they were planning to spend their time during lock down, most residents indicated that they would watch movies (75.4%), read content on social media (66.5%), read books (62.3%), read articles (61.4%) and work at home (52.9%) (Fig. 4). More males indicated that they would watch movies (75.7%), read content on social media (67.4%) and read articles (64.3%) than their female counterparts. More females than males indicated that they would read books (63.6%). Only a fifth (19.8%) of residents living in informal dwellings indicated that they would be working at home.

## Discussion

Voluntary self-isolation or quarantine is a well-established and proven containment measure for preventing the spread of highly infectious diseases in large populations during a pandemic outbreak, but it is only effective if properly implemented [28]. This paper examined perceptions and preparedness for self-isolation or quarantine and preparations for lockdown during the initial phase of the COVID-19 pandemic in South Africa. Overall, our findings demonstrate varied realities and readiness for isolation, quarantine and lockdown among South African residents, which varied by age, race, locality and other socio-economic factors. Self-isolation has also been previously shown to be directly impacted by socio-economic status, age, education and whether or not families have to care for vulnerable individuals like children or the elderly [29]. Additionally, these findings show that more than half of the respondents were prepared for self-isolation, child-isolation and the isolation of the elderly. Similarly, in a study conducted in Israel, a self-isolation compliance rate of less than 57% was reported from an online survey [30]. It is noteworthy to indicate that respondents living in informal dwelling settlements were the least prepared for isolation and quarantine mostly due to the stark inequalities of the South African society and with no space available for this in poor households.

With regards to the perception on possibility of ending up in self-isolation or quarantine, the likelihood of people reporting that they thought they might end up in self-isolation or quarantine were higher among other race groups compared to Black Africans and among those who reported moderate risk perception and high risk perception when compared to those with low risk perception for contracting the disease. The race disparities may be due to knowledge and awareness differences about the COVID-19 pandemic between other race groups and Black Africans. Surprisingly, older citizens (30 years and older) who are more vulnerable to contracting COVID-19, were less likely to think they might end up in self-isolation or quarantine compared to their younger counterparts. Those who were unemployed were less likely to think they might end up in self-isolation or quarantine compared to those employed in full time jobs. This may be attributed to the fact that initially, COVID-19 was perceived to be associated with international travel, of which the unemployed are not exposed to. The COVID-19 virus was therefore initially seen as the virus that attacks middle or working class and wealthy South Africans as they are the ones that normally travel abroad. This highlights the importance of communicating factual and rational information about COVID-19 in order to foster a positive social climate for communities to endorse healthy habits and to comply with the recommended preventive health behaviours.

Previous studies reported significant gender differences in the behaviours related to pandemics, especially in how these behaviours relate to self-isolation [14]. Our results also found that males were significantly more prepared for self-isolation or quarantine compared to females. Higher proportions of male citizens reported that they would be able to self-isolate or quarantine, and separate children and elderly from the rest of the family compared to their female counterparts. These differences based on gender may be an indication of the disparities in terms of resources between male headed households when compared to female headed households. On the contrary, there were no gender differences when it came to individual readiness for self-isolation [29]. Furthermore, respondents with differing socio-economic statuses had reported equally high personal readiness for the lockdown.

The results of the current study also show that higher age was associated with better preparedness. Those aged 40 years and above were more likely to be prepared to self-isolate, and separate children and the elderly from the rest of the family than those aged 18–29 years. Age has also been previously found to be a predictor of self-isolation readiness during a pandemic [29]. The odds of being prepared to self-isolate, or to separate children and the elderly from the rest of the family were higher among other race groups when compared to Black Africans. The likely reason for this could be that the Apartheid policies afforded Coloured, White and Indian/Asian sub-population groups more socio economic benefits in terms of housing, education and general wellbeing [31] . The socio-economic inequalities in South Africa are often visible across racial lines due to the historical legacies of legalized racial segregation [31–33].

When comparing employment status, this study found significant differences between the five employment categories for all three isolation scenarios. Zhang et al. [28] also reported significant association between socioeconomic status and preparedness for self-isolation. Self-employed residents showed the highest preparedness for all three forms of isolation when compared with people who are in employment which is to be expected as they have more personal control of their time. Those employed informally or part time were less likely to be prepared for child-isolation and elderly-isolation than those employed full time. On the contrary, a study in Israel found that self-employed individuals were reportedly more prone to refuse self-quarantine measures when compensation was removed, which emphasizes that socio-economic conditions and survival play a big role in the decision to comply with regulations [30].

Our study also found that residents of formal dwellings reported higher preparedness to self-isolate and to separate children and elderly from the rest of the family compared to residents of informal dwellings. This finding is not surprising as South Africa has one of the most unequal societies in the world, where 14% of households are located in informal settlements that have crowded squalid living conditions [34].

Panic buying is one of the common issues that accompanied the COVID-19 pandemic worldwide. Previous studies indicate that grocery purchases make up the largest component of shopping during periods of outbreaks [35]. In order to understand how South Africans reacted to this, residents were asked to indicate if they had bought more of the 14 listed essential items in preparation for the lock-down period with more than one option possible. Interestingly, toilet paper, which was the most commonly purchased product in many countries, was the fourth most purchased product among South African residents. Jung and colleagues’ utilized data from credit card purchases and was unable to list the specific items purchased [35]. Although panic buying of masks had been reported elsewhere in China (Hong Kong) and Italy [36], it didn’t make it to the top five most purchased items in South Africa.

The fact that more than half of the respondents indicated that they would be able to work remotely while at home showed that the country’s economy would not be on complete shutdown during the lockdown. This further indicated South Africans’ preparedness for lockdown but was also a clear demonstration of how socio-economic status can have an impact on how one would experience a national lockdown. The implications of the COVID-19 outbreak on the global economy and health care infrastructure mean that existing policies need to be reviewed to help mitigate the current crisis. The South African government had no choice but to institute a mandatory nationwide lockdown and forced the closure of businesses deemed as non-essential services [21]. The most immediate consequences of the lockdown included loss of income for businesses, loss of employment across multiple sectors and loss of tax revenue. The government should therefore concentrate on reducing the spread of the virus and channelling monetary relief towards the most vulnerable, including small and medium-size enterprises, households and informal workers [37, 38]. Although these measures can offer temporary relief, more long-term avenues need to be considered. For example, women constitute a subgroup of the vulnerable because they are at an increased risk of infection from COVID-19. This is largely due to women being the main caregivers in their communities, homes and health facilities [39]. Protection measures against increased exposure among vulnerable groups should be considered when designing health care infrastructure post COVID-19.

One of the strengths of the HSRC COVID-19 online survey was the unusually higher response rate from the White community in the formal established suburbs. The White community in South Africa is usually under-represented in most traditional national surveys that are conducted even by constitutional organizations such as Statistics South Africa. The key message from this survey is that the research community might need to consider the combination of the two (online and traditional) surveys going forward to address under-representation by White respondents in national surveys. The response rates may also be an indication of how seriously the COVID-19 pandemic was being taken by all of society regardless of their race or socio-economic status.

There are some limitations that should be considered in this study. The analysis was based on self-reported data and therefore may be prone to social desirability bias. Participants were recruited from the population who has access to technology and internet, which may have introduced selection bias. Relatively little is known about the characteristics of people in online communities. To minimize the impact of these limitations, the data was benchmarked to the general population utilizing Statistics South Africa’s 2019 mid-year population estimates, with the aim of correcting bias that may have resulted from the sampling strategy, and this allowed for generalizability of the findings to the South African population. Despite these limitations, this study is the first to reveal perceptions and preparedness of South Africans for self-isolation or quarantine during the COVID-19 pandemic.

## Conclusions

Generally, although a lower proportion of South African residents (40.1%) thought they might end up in self-isolation or quarantine, there were higher proportions indicating readiness or preparedness for self-isolation (59.0%), child-isolation (53.8%) and elderly-isolation (59.9%). This shows that the majority of residents were prepared for these public health and socio-behavioural interventions implemented by government in order to deal with the COVID-19 outbreak in the country. This study displayed the relevance of the social sciences in public health research during infectious disease outbreaks, as South Africa’s public health response relies very much on the behaviour of individuals, communities and society at large. Early indications and case number progression seemed to show that the country was flattening the curve due to the adoption of transmission-reducing behaviours. These occurred in the initial absence of large scale biomedical or pharmaceutical interventions such as large-scale community-based screening, testing and vaccine delivery.

## Supplementary Information

**Additional file 1.** Final COVID-19 HSRC Questionnaire.

## Data Availability

Data and materials are available from the lead author upon reasonable request.

## Abbreviations

COVID-19: Coronavirus Disease 2019
HSRC: Human Science Research Council
MERS: Middle East Respiratory Syndrome
SARS: Severe Acute Respiratory Syndrome
SARS Cov2 (COVID − 19): Severe Acute Respiratory Syndrome Coronavirus 2
Svy: Survey prefix command
WHO: World Health Organization
