# Supplementary material for: Preparedness for self-isolation or quarantine and lockdown in South Africa: results from a rapid online survey
Source: BMC Public Health. 2021 Mar 23;21:580. doi: 10.1186/s12889-021-10628-9 (PMC7987115; doi:10.1186/s12889-021-10628-9)
Supplement: Supplementary file 1 — Additional file 1. Final COVID-19 HSRC Questionnaire. [file 12889_2021_10628_MOESM1_ESM.pdf]

Quantitative Online Questionnaire:

Street talk" - South African Communities' Understanding of and response to the Coronavirus (COVID-19) Outbreak

1) INFORMED CONSENT

Dear Fellow South Africans,

Our country has recently joined the world in directly facing the threat of the COVID-19 pandemic. Individuals, communities and businesses have all been, and will in the immediate future be affected by the progression of this disease, in South Africa, across the continent and across the globe.

With schools closed, trade under pressure and uncertainty facing South Africans daily, the HSRC would like to ask you your views on the COVID-19 pandemic.

It is important that we collect and use such information for better preparedness and to reduce the impact of ongoing efforts by communities and government to stem the spread and provide the best information and social support to all citizens.

No personal information will be recorded and your response is COMPLETELY ANONYMOUS.

Should you wish, you may provide your contact number if you want us to contact you and discuss this research. You are however, in no way required to do so.

If you agree to participate in this survey, please respond to the questions and statements below. If you do not wish to participate, you may close this screen and no information will be recorded.

It should take you no more than 15 minutes to complete the survey.

Who to contact if you have been harmed or have any concerns

This research has been approved by the HSRC Research Ethics Committee (REC). If you have any complaints about ethical aspects of the research or feel that you have been harmed in any way by participating in this study, please call the HSRC's toll-free ethics hotline 0800 212 123 or e-mail research.ethics@hsrc.ac.za .

Please contact Dr Saahier Parker if you have any queries about this research (082 928 7473, OR Sparker@hsrc.ac.za).

- **I agree to voluntarily provide information related to my understanding and sentiment surrounding the COVID-19 emergency.**
- **I acknowledge that should I wish to withdraw from this research at any point I may abandon completing the short survey questionnaire and close this screen with no information recorded.**
- **I will not be asked to identify myself in anyway and all my responses are completely anonymous.**

STATEMENT	ACTION
I provide informed consent and agree to participate	Proceed to QUESTION 2
I do not provide consent and do not agree to participate	Close survey (no information recorded)
I would like to be contacted in this regard	Proceed to QUESTION 42

2) Have you heard about "**COVID-19**" or "the **COVID-19 virus**" or "**Coronavirus**"?

**YES, I have heard about it**	**NO, I have not heard about it**	**I DON'T KNOW**

3) COVID-19 is caused by

**BACTERIAL INFECTION**	**INSECT BITE**	**ANIMALS**	**VIRAL INFECTION**	**I DON'T KNOW**

4) COVID-19 is spread by direct contact with the virus from: (Select all that apply)

**INFECTED PERSONS COUGHING OR SNEEZING**	
**PETS**	
**BY BEING IN A PUBLIC GATHERING WHERE THERE IS AN INFECTED PERSON**	
**VIRUS CONTAMINATED SURFACES**	
**TOUCHING YOUR FACE AFTER YOU HAVE BEEN IN CONTACT WITH AN INFECTED PERSON**	
**I DON'T KNOW**	

5) After how long will an infected person show signs of being sick?

**IMMEDIATELY**	**AFTER 1-2 DAYS**	**AFTER 2-14 DAYS**	**AFTER 15-20 DAYS**	**I DON'T KNOW**

6) Which of the following best describes the symptoms of COVID-19: (Select all that apply)

**BODY PAIN**
**SWEATING**
**SHORTNESS OF BREATH**
**HEADACHES**
**COUGH**
**RUNNING NOSE**
**SNEEZING**
**RED-ITCHY EYES**
**FEVER**
**I DON'T KNOW**

7) Prevention of COVID-19 infection is best achieved by (Select all that apply)

**COVERING YOUR MOUTH WITH A FLEXED ELBOW WHEN COUGHING**
**STAYING AT HOME DURING THE LOCK DOWN**
**USING GLOVES**
**USING FACE MASK**
**WASHING YOUR HANDS REGULARLY FOR 20 SECONDS**
**OBTAINING A VACCINE**
**I DON'T KNOW**

8) How do you rate your **PERSONAL RISK** of contracting COVID-19

**VERY HIGH RISK**
**HIGH RISK**
**MODERATE RISK**
**LOW RISK**
**VERY LOW RISK**

9) Why do you believe that you are at the **SELECTED LEVEL** of risk? (Select all that apply)

**I AM IN A YOUNG AGE GROUP**	
**I HAVE UNDERLYING MEDICAL CONDITIONS**	
**I WASH MY HANDS REGULARLY**	
**I SMOKE**	
**I WORK IN A HIGH-RISK ENVIRONMENT** (Hospital, Police Station, Essential Services)	
**MY HOME ENVIRONMENT PLACES ME AT RISK**	
**I AM SELF-ISOLATING**	
**USING GLOVES**	
**BECAUSE I AM STAYING HOME DURING THE LOCK DOWN**	
**I AM IN A HIGH-RISK AGE GROUP**	
**I AM GENERALLY HEALTHY**	
**I USE A FACE MASK**	
**WE ARE ALL AT RISK**	

10) In your opinion, do people in these categories have a **HIGHER** or **LOWER** risk of contracting COVID-19 than you, personally:

	HIGHER THAN MY PERSONAL RISK	ABOUT THE SAME RISK	LESS THAN PERSONAL RISK
THE WORLD			
SOUTH AFRICA			
MY PROVINCE			
MY NEIGHBOURHOOD			
MY FAMILY			

11) Do **YOU** Currently SMOKE or use the following?

	Yes, daily	Yes, less than daily	No, not at all
SMOKING TOBACCO (cigarettes, pipes, cigars)			
VAPES/ ELECTRONIC CIGARETTES			
SNUFF, CHEWING TOBACCO			
HOOKA PIPES			

12) Do **OTHER PEOPLE** in your house Currently SMOKE or use the following?

	Yes, daily	Yes, less than daily	No, not at all
SMOKING TOBACCO (cigarettes, pipes, cigars)			
VAPES/ ELECTRONIC CIGARETTES			
SNUFF, CHEWING TOBACCO			
HOOKA PIPES			

13) Do you feel each of the following are able to manage the South African COVID-19 outbreak?

	YES	NO	DON'T KNOW
SOUTH AFRICAN HEALTH SYSTEM			
NATIONAL GOVERNMENT			
PROVINCIAL GOVERNMENT			
LOCAL DOCTOR			
YOUR LOCAL CLINIC / HOSPITAL			
YOUR LOCAL SUPERMARKET			
YOUR FRIENDS / FAMILY			

14) Do you think you may end up in a situation of **SELF-ISOLATION** or **QUARANTINE**

**YES**	**NO**	**DON'T KNOW**

15) If **QUARANTINE / SELF-ISOLATION** should become necessary, does your home have a separate space for you to do so?

**YES**	**NO**	**DON'T KNOW**	**N/A – I LIVE ALONE**

16) If there are **CHILDREN** in the home needing self-quarantine, would you be able to separate them from the rest of the family? (Aged 0-14)

**YES**	**NO**	**DON'T KNOW**

17) If there are **ELDERLY MEMBERS** in the home needing self-quarantine, would you be able to separate them from the rest of the family?

**YES**	**NO**	**DON'T KNOW**

18) Have you been doing any of the following **DURING THE PAST WEEK** as a result of the COVID-19 emergency? (Select all that apply)

**USING FACE MASKS**	
**COVERING COUGHS OR SNEEZES WITH A TISSUE OR FLEXED ELBOW**	
**WEARING HAND GLOVES**	
**USING HAND SANITIZER**	
**WASHING MY HANDS MORE FREQUENTLY**	
**STAYING IN MY HOUSE and DECREASED MY SOCIAL INTERACTION**	
**SELF-ISOLATING**	
**OTHER**	
**I HAVEN'T TAKEN ANY PRECAUTIONARY PROCEDURES YET**	

19) Please indicate if you have bought **MORE** of the following items in preparation for the lock-down period? (Select all that apply)

**CANNED / DRIED FOODS**
**DRINKING WATER**
**HYGIENE SUPPLIES TOILETRIES / PERSONAL HYGIENE PRODUCTS**
**CLEANING SUPPLIES**
**MEDICAL SUPPLIES**
**LUXURY ITEMS / SNACKS / TREATS**
**TOILET PAPER**
**NAPPIES**
**MEDICAL GLOVES**
**FACE MASKS**
**HAND SANITIZER**
**MEAT / FRESH PRODUCE**
**FUEL / PARAFFIN / GAS / BATTERIES** (heating, cooking or lighting)
**HAVE NOT STOCKED UP ON ANYTHING**

20) Which of the following best describes your home?

**STAND ALONE HOUSE**	
**TOWN HOUSE / SEMI DETACHED HOUSE**	
**FLAT IN BLOCK OF FLATS**	
**TRADITIONAL DWELLING / HUT**	
**HOUSE / ROOM IN BACK YARD**	
**SHACK IN BACK YARD**	
**INFORMAL DWELLING / SHACK NOT IN BACK YARD**	
**TENT / CARAVAN**	

21) Now that South Africa is in a mandatory lock-down, how would you occupy your time for 21 days?

**READ BOOKS**	
**WATCH MOVIES / TV**	
**READ ARTICLES ON THE INTERNET**	
**SOCIAL MEDIA – READING**	
**SOCIAL MEDIA – POSTING CONTENT**	
**INVITE FRIENDS HOME**	
**GARDENING**	
**SLEEPING**	
**STUDYING**	
**WORKING AT HOME**	
**SPENDING TIME WITH FAMILY**	
**I REALLY DON’T KNOW WHAT I WILL DO**	
**HAVE NOT CONSIDERED WHAT I WOULD DO**	

22) How much do you feel you know about the COVID-19 pandemic?

**I KNOW WAY LESS THAN I SHOULD KNOW**
**I KNOW A LITTLE, BUT NOT ENOUGH**
**I KNOW ENOUGH**
**I KNOW A LITTLE MORE THAN MY FRIENDS**
**I AM UP TO DATE ON THE LATEST RESEARCH**

23) Where do you get most of your information on COVID-19? (Select all that apply)

**LOCAL TELEVISION**	
**SATELLITE TELEVISION** (Dstv, OpenView)	
**RADIO**	
**PRINT NEWSPAPERS**	
**WHATSAPP**	
**SOCIAL MEDIA** (Excluding WhatsApp)	
**NEWS WEBSITES or MOBILE APPS**	
**GOVERNMENT SOURCES (President; Minister Of Health etc.)**	
**SPOUSE or CHILDREN**	
**PERSONAL DOCTOR**	
**FRIENDS**	
**FAMILY**	
**OTHER MOBILE CHAT SERVICES**	
**EMAIL**	
**SMS**	
**SCIENTIFIC JOURNALS**	

24) How much do you **TRUST** each of the following sources of information on COVID-19?

	LOW TRUST	MODERATE TRUST	HIGH TRUST
**LOCAL TELEVISION**			
**SATELLITE TELEVISION** (Dstv, OpenView)			
**RADIO**			
**PRINT NEWSPAPERS**			
**WHATSAPP**			
**SOCIAL MEDIA** (Excluding WhatsApp)			
**NEWS WEBSITES or MOBILE APPS**			
**GOVERNMENT SOURCES (President; Minister of Health etc.)**			
**SPOUSE or CHILDREN**			
**PERSONAL DOCTOR**			
**FRIENDS**			
**FAMILY**			
**OTHER MOBILE CHAT SERVICES**			
**EMAIL**			
**SMS**			
**SCIENTIFIC JOURNALS**			

25) I believe the threat from the COVID-19 is exaggerated in the media.

STRONGLY AGREE	AGREE	NEUTRAL	DISAGREE	STRONGLY DISAGREE

26) I feel there is way too much information in the media and I can't keep up with it all

STRONGLY AGREE	AGREE	NEUTRAL	DISAGREE	STRONGLY DISAGREE

27) What Information do you want to receive about COVID-19?

**PLACES WHERE THE CASES ARE IN SOUTH AFRICA**	
**NUMBER OF PEOPLE INFECTED IN SOUTH AFRICA**	
**WHAT THE GOVERNMENT IS DOING ABOUT COVID-19**	
**HOW TO PREVENT MYSELF FROM GETTING INFECTED**	
**WHAT HAPPENS IF I GET INFECTED**	
**HOW TO KNOW IF ONE IS INFECTED**	
**WHO IS MOST AFFECTED BY THE VIRUS**	
**WHAT MY LOCAL CLINIC WILL DO FOR ME**	

28) This whole COVID-19 crisis will be over in the next:

**FEW DAYS**
**FEW WEEKS**
**FEW MONTHS**
**WILL LAST AT LEAST 12 MONTHS**
**I DON'T KNOW**

29) When thinking about the Coronavirus here in South Africa, which of the following do you think is most likely to happen over the **NEXT MONTH**?

WE WILL BE OVER THE WORST OF IT - THINGS WILL BEGIN TO IMPROVE	
THE SITUATION WILL REMAIN LARGELY THE SAME AS IT IS NOW	
THE WORST IS YET TO COME – THINGS WILL START TO GET WORSE	
I DON'T KNOW	

30) Is there currently a **CURE** or **MEDICATION** to treat COVID-19?

YES	NO	DON'T KNOW

31) Governments and pharmaceutical companies will **DEVELOP A VACCINE** within:

THE NEXT 6 MONTHS
WITHIN ONE YEAR
AFTER THE NEXT 12 – 18 MONTHS
I DON'T KNOW

32) Foreign nationals arriving by land, air or sea, **TESTING POSITIVE** for COVID-19 should be sent back to their home country immediately without exposing the South African population.

STRONGLY AGREE	AGREE	NEUTRAL	DISAGREE	STRONGLY DISAGREE	Elect not to answer

33) If you were to be quarantined at home, would it affect your income?

YES, A LOT	YES, A LITTLE	NO

34) If you are employed, do you feel your employer had done enough to protect you in the workplace against transmission of COVID-19? (e.g. provide hand sanitisers, flexible working arrangements)

YES	NO

35) Are there school-going children in your household?

YES	NO

36) Are there any students attending tertiary institutions in your household?

YES	NO

37) Do you think the closing of schools and restriction of public gatherings and sports events will slow the spread of the virus?

YES	NO	Don't know

38) Do you PERSONALLY know anyone who has tested for COVID-19?

YES	NO

39) What is your relationship to those individuals? (Select as many as appropriate)

NOT APPLICABLE	
FAMILY	
FRIEND	
NEIGHBOUR	
PEOPLE AT WORK	
CHILD	
PARTNER	

40) Have you personally been tested for COVID-19?

YES	NO

41) If you do start showing symptom and you suspect you may have been exposed to COVID-19, what would be your immediate course of action?

ISOLATE MYSELF AND CALL THE COVID-19 HOTLINE	
POST THE NEWS TO MY SOCIAL MEDIA	
ALERT MY EMPLOYER	
AVOID MY PETS	
TREAT IT LIKE I WOULD ANY FLU	

42) Do you subscribe to any of the following **GENDER** groups?

FEMALE	MALE	OTHER	None

43) Please indicate which **AGE GROUP** you currently fall into:

18 – 29 YEARS OLD	30-39 YEARS OLD	40 – 49 YEARS OLD	50-59 YEARS OLD	60-69 YEARS OLD	OLDER THAN 70

44) To which **POPULATION GROUP** do you belong

BLACK	WHITE	COLOURED	INDIAN / ASIAN	NON-SOUTH AFRICAN

45) Which **PROVINCE** do you live in?

Western Cape
Eastern Cape
Northern Cape
North West
Free State
KwaZulu-Natal
Gauteng
Limpopo
Mpumalanga

46) Have you travelled out of the country in the **LAST 30 DAYS**?

**YES**	**NO**

47) What is your current **EMPLOYMENT STATUS**?

Employed – full time (fixed salary per month)
Employed –informal sector/ part time (non-fixed salary per month)
Unemployed
Home Duties
Full Time Student
Retired
Self Employed

48) Please provide us with your **CELL PHONE NUMBER** (OPTIONAL)

